# Pulp Canal Filling

**Published:** 1895-01

**Authors:** Mark G. McEelimney

**Affiliations:** Ottawa.


ARTICLE VI.

### PULP CANAL FILLING.

BY MARK G. MCEELIMNEY, D. D. S., OTTAWA.

Almost every dental journal contains an article, and every dental association has a section, pertaining to the filling of root canals.

It is through this interchange of ideas that we arrive at the best methods of accomplishing our objects.

While my way of doing this is by no means new, I think that a description may be interesting.

After destroying the nerve, the first and most important thing is cleanliness. Every possible particle and fibre of dead tissue must be removed, and the canals made thoroughly antiseptic.

I say every possible particle, because it is impossible for the most skilled operator to remove everything, except in very accessible cases. The difficulty increases with the remoteness and tortuousness of the canal.

There is a tendency amongst dental writers to forget the practical limitations, and construct a very fine theory of perfect work on paper.

While I recognize fully the absolute necessity of a high standard of excellence, toward which we should constantly struggle, there is also present to me the fact, that the very best work in the treatment of canals depends upon the forms of the canals themselves.

I have split open many extracted teeth, and in a comparatively large proportion have found, that the canals were so constricted that theoretically, perfect treatment would be a very difficult matter.

In cases where nerve broaches cannot be used with full satisfaction, it is necessary to depend for success upon thorough medication of the remaining debris.

For removing debris from canals, the prepared nerve bristles are very unsatisfactory. They are either too soft or too brittle, or the barbs rub off very easily.

It is a wonder that so many firms keep on making and so many dentists buying, articles that are of no earthly value to anyone who wishes to do thorough work.

A broach must be stiff, springy, and of even taper. It must also be cheap and adaptable to various circumstances. Broaches for lower molar roots shoud be somewhat flattened to suit the canals. The points must be very sharp to avoid pushing debris ahead; while the butt must be of sufficient thickness to give strength.

To make nerve broaches that will do reasonably satisfactory work, take No. 18 piano wire, and draw the temper a little.

A little experience will enable one to draw to any desired temper for any particular case. A Bunsen flame will do very well. The wire should be left sufficiently stiff to come back straight when the point is deflected thirty or forty degrees. Place a piece of hard wood end up in the vise, and with the corner of a file cut a light groove parallel with the jaws. This groove is to keep the wire from slipping while it is being filed.

Place the piano wire in a pin vise and file it taper with a square section, or slightly flattened for some cases. Care must be taken to make the taper even from butt to point, or the broach will be liable to break.

With a sharp graver nick the square corners of the broach, and the result is a series of barbs that will stand a great deal of use. The barbs may be placed on one, any or all corners, according to requirement. The butt must be adapted to the particular kind of broach-holder used.

For a screw chuck-holder the butt should be made square. For a sliding ring-chuck the butt will stay better if slightly flattened.

A foot of piano wire will make nine broaches, and costs less than a cent. One of these broaches will do more work than three ordinary ones at fifty cents a dozen.

Having now a serviceable broach, it is necessary to use it rightly. It should not be sent to the apex at first, but the debris must be carefully removed as one goes up, care being taken to avoid wadding the narrower portion with debris, as it is very difficult to remove and may get solid enough to be taken for the apex. To properly cleanse a root requires time and patience, for it is a really tedious operation.

Having removed all dead matter that will come out, the next step is the thorough medication of the root. A few fibres of cotton twisted around a fine broach makes a most efficient pump. Each dentist has a favorite preparation for roots—suffice it to say that whatever is used must be used thoroughly.

Having the canals clean and ready to fill, I do not dry them; but rather leave them full of root preparation, which is forced into every crevice by the filling.

The most important question is, What material for filling? When a tooth is healthy and the indications favorable, I use silver.

Even where there has been periosteal trouble, now cured, or even an abscess that has yielded to treatment, I recommend silver as the most impervious and safest material. Silver can be got into more difficult situations, and is surer to stay, than any other material. Silver is less likely to be affected by adverse circumstances in the canal; moisture and constriction do not prevent it from reaching its place.

In the case of cement, moisture is fatal to the proper pumping of the material; and I doubt if one dentist in a hundred can get one canal in a hundred fit to hold cement.

Another objection to cement is that subsequent leakage through the apex must eventually disintegrate it in the immediate vicinity, and, once disintegration is set up, there is an end to all confidence.

The silver can be put into position by means of pluggers of various sizes made of piano wire and used in the broach-holder.

Filling a canal with silver is at first somewhat difficult, and never becomes really easy. The best way is to put a pellet in the pulp-chamber, and with a burnisher press it gently against the canal openings, then send it up the canal with the probe-like plugger, the end of which should be cut off square. This cuts a cylinder out of the pellet and carries it ahead right up the canal. Whatever small space is beyond is forced full of root preparation, which should be crystallizable, astringent and antiseptic.

In cases where there has existed chronic abscess, and a recurrence is feared, where it may some day be necessary to reopen the canal, I insert a gutta-percha point dipped in chloroform. These points I make by rolling gutta-percha pellets on a glass slab with a warm spatula. They can be rolled to any desired length, thickness or angle. While I do not regard gutta-percha as equal to silver, yet it is the next best material where a non-removable filling is distinctly contra-indicated.

It has been suggested to saturate the dead pulp with some powerful agent, and leave it in the tooth. This is little less than criminal.

The use of cotton saturated with preservaties as a canal filling I regard as unscientific, careless, and the invention of a lazy man, whoever he may be. If there is such a thing as leakage through the apical opening, the preservative must in time wash out; then the canal is left filled with unprotected organic matter. Disintegration, irritation and abscess are only a matter of time. At college I learned that cotton-filling was good for roots. For the first few months all went well with the few teeth that a cold and unsympathetic public allowed me to fill. Then the chickens "came home to roost." Those chickens are all disposed of some time ago, and I have learned a valuable lesson, and that is, that a nice, easy way of doing a difficult operation is generally no good. There is no royal road to successful root-filling; it is a test of ability.—*Dominion Dental Journal.*

---

